# Following in Jakobson and Lévi-Strauss’ footsteps: A neurocognitive poetics investigation of eye movements during the reading of Baudelaire’s ‘Les Chats’

**DOI:** 10.16910/jemr.13.3.4

**Published:** 2020-05-11

**Authors:** M Fechino, A M Jacobs, J Lüdtke

**Affiliations:** 1 Aix-Marseille University and CNRS, France; 2 Center for Cognitive Neuroscience, Berlin (CCNB), Freie Universität Berlin, Germany; 3 Freie Universität Berlin, Germany

**Keywords:** Eye movement, reading, poetry, neurocognitive poetics, visual presentation

## Abstract

Following Jakobson and Levi-Strauss [1] famous analysis of Baudelaire’s poem ‘*Les Chats’ (‘The Cats’)*, in the present study we investigated the reading of French poetry from a Neurocognitive Poetics perspective. Our study is exploratory and a first attempt in French, most previous work having been done in either German or English (e.g. [2] [3] [4] [5] [6]). We varied the presentation mode of the poem *Les Chats* (verse vs. prose form) and measured the eye movements of our readers to test the hypothesis of an interaction between presentation mode and reading behavior. We specifically focussed on rhyme scheme effects on standard eye movement parameters. Our results replicate those from previous English poetry studies in that there is a specific pattern in poetry reading with longer gaze durations and more rereading in the verse than in the prose format. Moreover, presentation mode also matters for making salient the rhyme scheme. This first study generates interesting hypotheses for further research applying quantitative narrative analysis to French poetry and developing the Neurocognitive Poetics Model of literary reading [NCPM; 2] into a cross-linguistic model of poetry reading.

## Introduction

*‘Verse’ is not the same as ‘poetry’. We say that a text is ‘verse’ when it is divided into lines. But in contrast, calling a text ‘poetry’ is a way of valuing it, saying that it offers something experientially special.* [7]

French poetry developed through centuries, from Turold’s *La chanson de Roland* to Prévert’s *Barbara* including Hugo’s *Demain dès l’aube* and Ronsard’s *Mignonne, allons voir si la rose*. There are so many examples and diversities but there is a general agreement that poetry is specifically well suited to create emotions and aesthetic feelings [cf. 8]. However, there seems to be no empirical study yet which uses French poetic texts to investigate the affective-aesthetic experiences and responses accompanying poetry reading from a cognitive psychology point of view. Based on the Neurocognitive Poetics perspective [2], the aim of this study is to understand the interaction between a formal textual surface feature (i.e., visual presentation mode) and specific word level features of a poem (e.g., rhyme scheme, parallelisms, metaphors, etc). Following proposals by Jacobs [9] [10] and Dixon and Bortolussi [11] regarding the methodology applied in scientific studies of literature, in addition to a direct offline method (i.e., questionnaires) we also used an indirect online method (i.e., eye tracking) to assess processes taking place during initial reading and comprehension.

### Poems as a study paradigm

The use of literary texts represents an innovative expansion in cognitive studies. It is relatively new having participants read large portions of literary texts. This is part of both the Cognitive and Neurocognitive Poetics perspectives [12] [13]. It is a challenge for experimental approaches [14] [15] [16] [17] [18], though, both theoretically and methodologically. Theoretically, because of a lack of appropriate models allowing to accurately predict reader responses, and methodologically, because new methods require to deal with the multitude of stimulus variables (i.e., text features) that potentially influence reading experience and behavior. Nevertheless, there are already various examples of neurocognitive studies using longer literary texts as stimuli, for example long sections of prose [19] [20] [21], proverbs [22] [23], or poetry [5] [6] [24] [25] [26] [27] [28] [29]. Here, we want to understand the processing of poetry, and therefore it is necessary to present not only text parts but to use an entire poem with a lot of rhetorical figures on all levels shared between lines, i.e. the famous poem *Les Chats*.

Literary texts are based on artistic techniques and tools which distinguish them from other human creations. The whole meaning of poetry only becomes clear when using the complete text and not some isolated stanzas. Our choice of the sonnet *Les Chats* was motivated by a famous article of Jakobson and Lévy-Strauss [1] which can be considered as the prototype of a quasi-quantitative text analysis of a poem at four relevant text levels: metric, phonological, morpho-syntactic and semantic. It allows to develop a transparent code and tools responding to scientific rigor [30]. The authors’ structural analysis of *Les Chats* [31] was based on Jakobson’s extension of Bühler’s [32] organon model of language, the ‘new organon’ which offers six functions instead of the three original ones, the ‘poetic function’ being the most relevant for the present purpose. This poem represents a good basis for an experimental study given that the text was already analysed in a quasi-quantitative way raising a controversy among literary scholars of the time (e.g. [33] [34] [35] [36] [37]). Thus, for the neurocognitive poetics perspective, Jakobson and Lévy-Strauss’ [1] article presents a ‘model’ for formally analysing poetry. Thus, the NCPM combines Jakobson’s four levels of text analysis (metrical, phonological, morpho-syntactic, semantic) with four feature types (sublexical, lexical, inter-lexical and supra-lexical). Moreover, sonnets have a rigid structure allowing to compare them more easily with each other thus facilitating the investigation of cognitive and affective-aesthetic processes [38] [39]. However, Jakobson and Lévi-Strauss used a quasi-quantitative method to describe a lot of features on different levels but not all their analyses are complete for the whole poem, and there are missing details on how they selected and identified the features. This led to further questions about how to use this incomplete information, but also and more significantly, how to combine those features at different levels. This question is still an open task for future research in the neurocognitive poetics framework.

### Aim and rationale of the present study

In the scientific study of literature, four basic methodological approaches can be distinguished on the basis of the absence/presence of either a text manipulation and the application of indirect online methods (see Tables 1 in both [9] [11]). Here we chose a combination of two standard approaches: the experimental manipulation of a formal textual feature (presentation mode) of a poem while leaving the original text unchanged. We specifically focused on an indirect online method of investigation, eye tracking, to assess both experiential and behavioral aspects of the reading act.

Especially for printed poetry, the visual presentation is important and meaningful: the visuo-spatial (graphic) configuration of the text is not randomly defined as in space poetry for example (e.g., Apollinaire’s *Calligrammes*), and authors typically decide which visual presentation best suits their aims [8]. However, as expressed in the initial quote by Fabb [7] lineation itself does not make a text poetic, although it may facilitate certain aesthetic effects, while challenging the integration of the meaning of sentences that are often spread across several lines [4]. Explaining how readers process, understand and appreciate poetry is also a challenge to cognitive psychology because of its usually ‘crazy syntax’, i.e. the reordering or omission of elements in ways not legitimate in ordinary language. Thus, Fabb [7, p. 5] speculates that the usual hierarchical organization of a sentence (i.e., the tree structure) is destroyed by lineation, ‘the selection of compositional units in principle being unconstrained and eclectic (and thus not necessarily dependent on meaning)’.

### Presentation Mode

Several authors have worked on the question of poetry visualization. First, readers seem to use this information to determine if the text is part of the poetic genre, i.e. at least a part of potentially observed differences between prose vs. verse presentation can be considered as a top-down genre effect [40] [41]. The linguistic and visual features helping this categorization have been termed ‘signposts’, i.e. specific elements within the text considered to be meaningful by the reader, because of the pattern formation rules of a specific interpretive community [40] [42]. In addition to the effect on text categorization, presentation mode has also an effect on memory [43], facilitating the recall of rhetorical features [44].

Theoretically, at the surface level, the distinctive graphic form of a poem on a printed page or screen will produce a special perception-attention space for and in the reader [cf. 45]. Compared to prose, this space is smaller, well-structured and offers linguistic information ideally packaged for readers’ working memory [8]. Lines are defined as the fundamental unit of metered poetry [46] as its linguistic rhythm is in phase with the basic acoustic rhythm of 3 seconds – they measured 3.8 seconds in mean for reading French alexandrines. Specifically, for sonnets, ten syllables are usually distributed across 6 – 10 words (and roughly 114 words per sonnet, cf. [38], which makes a sonnet line cultural quasi-universal [46] [47]. It can be assumed that when presenting a Shakespeare sonnet in prose instead of its canonical 4+4+4+2 format, it would be processed very differently both at the behavioral level of eye movement patterns and the internal levels of ‘ception’ (i.e., derived from the word “ceptio”, a generalization of perception and conception; [48] [49]). This very likely will lead to a less efficient cognitive processing, a different attention resonance [18] and an overall diminished affective and aesthetic response. Inspired by the finding that poems presented this way received lower poeticity judgments [40], here we wanted to submit this prediction to further empirical testing including eye tracking data.

Two hypotheses have been put forward to explain this effect. Either the graphic form allows to create a visual frame that helps remembering the poem’s internal structure, or there could be a specific process when reading verse leading to focus on different text features than when reading prose. The second hypothesis has been supported by data from different studies showing longer reading times, better memory for surface information, but poorer memory for situational information when identical texts are presented as articles as opposed to stories or poems [50] [51] [52].

Moreover, the spatial organization of a poetic text (space management) makes parts of the text (e.g., words, verses, stanzas) more salient and thus facilitates their affective-aesthetic and cognitive processing [53]. Thus, space management can be considered a secondary punctuation system which influences the temporal syntactic structure [54]. Spaces could allow readers to express their own imagination and experiences [55], and may lead gaze direction to construct sentence meaning [56]. When reading prose, spaces do not carry much significance beyond signalling words and sentences separation and thus focusing on them does not help comprehension processes or even could lower cognitive fluency [57]. Space management has been assumed to increase aesthetic effects of written poetry, and led to the emergence of a space theory [58]. Precisely, Mallarmé, in *Un coup de dé jamais n’abolira le hasard (A Throw of the Dice will Never Abolish Chance,* [59]), explained in his foreword section that visual factors should be considered for interpretation. Space management could be linked to Gestalt theory as well. Thus, Tsur [60] stated that lines can be perceived as perceptual wholes (gestalts), if they can be contained in working memory, which functions in the acoustic mode like an echo box. Similarly to Turner and Poeppel [46], Crystal [61] saw the iambic pentameter line as optimal for neuronal processing: due to working memory limitations, five stressed syllables are the maximum people can comfortably handle within a single rhythm/tone-unit (but see [62] for a critique of this position). Baudelaire himself gave scrupulous attention to his poetry presentation during the printing process (printer’s proof annotated by the author, [63]). Space is undeniably a central feature for many poets in different cultures and should be considered as a main factor when studying verse specific reading.

Regarding eye tracking, an unpublished study by Fischer and colleagues [64, cf. 25] showed that for the same poem presented in prose vs. verse, readers developed different reading patterns: gaze duration was increased for the verse version with more fixations, shorter progressions and more rereading than in the prose version. This suggests that surface features shape the eye movement pattern to some extent, but it remains unclear to what extent lexicosemantic and other text features contribute to or interact with this presumable surface feature effect.

According to different studies, one key feature for poetry is the rhyme scheme. It is considered as a voice-punctuation of the poem [38] and is a sound foregrounding (FG) that attracts easily readers’ attention and contributes to the poem’s musicality [65 p. 367]. FG includes all kinds of text defamiliarization and is considered as a deviation from common language [44] [66] [67]. Nevertheless, to understand how poetry is processed and understood, it is not enough to focus on features of the text. According to Hanauer [68], the central step is the dynamic relationship between reader and text. Precisely, both rhyme and visual presentation are salient textual features that weigh massively in poetry distinction, it is an empirical question how both features influence the processing. Hanauer [68] also questioned how attention resources are used while reading poetry. Carminati et al. [25] asserted that when readers are confronted with a text they will make a decision depending on the text features, the context they are in and the reading goal on how they will pay attention to the text and on what. This is also led by readers’ knowledge assets.

Hence, different studies show the impact of both of these textual features on poetry reading. By manipulating visual and phonological variables, Hanauer [40] showed that both of these features impacted categorization judgments of poeticity with higher poeticity ratings for the version displaying highly visible features. Van Peer [44] showed better remembrance when the FG feature rhyme scheme was preserved. In the same path, recalls from the poem were better when the original verse presentation was unchanged [43].

### Hypothesis

*Visual Presentation processing of verse vs. prose:* Inspired by an idea on poetry production proposed by Fabb [7], we hypothesize that in poetry *reception* as well verse is translated in the brain into a sort of prose variant to facilitate comprehension of the ‘crazy syntax’ and obscure semantics of poetry. While this ‘translation’ process may take time, given that in ordinary language processing the sentence is the basic unit of semantic integration – while it is *the line* in poetry reading [7] [8] [12] [46] –, the prose version of our poem should be easier to read than the verse version, since it offers a more transparent sentence structure. In accordance with the aforecited previous research, we thus expect that the visual presentation of the poem *Les Chats* as a prose text will lead to shorter total reading times compared to a visual presentation in its originally verse form. If the global processing is indeed easier and faster in the prose compared to the verse presentation mode, this processing difference should be reflected also at the lexical level. So, we expected on average shorter total reading times for all words presented in the prose form compared to the verse form. Whether these differences come from an early facilitation and more automatic processes reflected in first fixation duration or gaze duration or from later processes associated with deeper comprehension and interpretation reflected in rereading, or in both, is an open empirical question.

*Rhyme scheme processing:* Empirical studies using offline measures like Hanauer’s early work showed that rhyme scheme influences the processing of poetry at different levels. Hanauer assumed that the initial choice of an appropriate reading strategy is based on the visual presentation but also on the presence of a rhyme scheme. Following ‘space theory’ it can be assumed that the visual presentation in verse form is the key that makes rhyme scheme a salient feature. Usually, rhyme words are presented at the very end of a line, while there are also possible internal rhymes which are not always systematic and are less visible. Therefore, we assume an interaction between rhyme words and visual presentation. Rhyme words should be processed longer compared to all other words, while this difference should be more pronounced in the verse compared to the prose presentation. This should lead to longer total fixation times but only when the rhyme scheme is salient. This effect should go beyond the usual finding of prolonged fixations on the last word of a line. As demonstrated in studies using prose textoids, the final word in sentences is associated with longer processing (e.g., [69], possibly due to semantic integration efforts (i.e., sentence wrap-up). Since, at the end of each line the reader has to prepare a line sweep this could further contribute to prolonged processing times. To control for these possible effects, we added an additional variable differentiating between the last words on a line in the prose version and all other words. If indeed the prolonged reading times for words at the line end were mainly due to wrap-up processes and preparations of a line sweep, this effect should also be visible in longer reading times for last words in the prose condition, compared to all other words. In contrast, in the verse presentation where the last words from the prose version appear somewhere in the middle of a line, no such effect should occur. Analogously, prose presentation where the last words from the verse version (i.e., rhyme scheme words) appear randomly in the text, no such effect should occur.

## Methods

### Participants

Eighteen native French participants (9 women; M_age_=31 years, SD_age_=13.94; age range: 19-70 years) were recruited from an announcement released at Freie Universität Berlin. All participants had normal to corrected-tonormal vision. All participants were naïve to the purposes of the experiment. Twelve participants were students. Participants gave their informed, written consent before beginning the experiment and were offered to participate to a lottery with 3 gift cards to win (10 euros each). This study was conducted in line with the standards of the ethics committee of the Department of Education and Psychology at Freie Universität Berlin.

### Apparatus

Participants’ eye movements were recorded with a sampling rate of 1000 Hz, using a remote SR Research Eyelink 1000 desktop-mount eye tracker (SR Reasearch Ltd., Mississauga, Ontario, Canada). Stimulus presentation was controlled by Eyelink Experiment Builder software (version 1.10.1630, http://www.sr-research.com/experiment-builder). Stimuli were presented on a 19-inch LCD monitor with a refresh rate of 60 Hz and a resolution of 1,024 x 768 pixels. A chin-and-head rest was used to minimize head movements. The distance from the participant’s eyes to the stimulus monitor was approximately 65 cm. We only tracked the right eye. Each tracking session was initialized by a standard 9-point calibration and validation procedure to ensure a spatial resolution error of less than 0.5° of visual angle.

### Design

We used a within-subject design with each participant reading both versions of the poem, the order being counterbalanced. We used those five different independent variables: Visual Presentation, Verse Last-Word, Prose LastWord, Reading Session, Word Length. Visual Presentation is verse and prose visual presentation presented in the hypothesis. Verse Last-Word is a binary variable representing whether a word is part of the rhyme scheme or not. To counterbalance that variable, we added Prose Last-Word which compares the last word of each line in the prose version to all other words. We added Reading Session (first vs. second reading) because due to the experiment design there is a global facilitation when reading two times the same text, and thus also with eye tracking data. The same text is reflected in the eye fixation patterns with shorter fixations and fewer regressive eye movements [6] [70] [71] [72]. We also add Word Length as it is one of the most important predictor of eye movements [73] [74] [75] [76] [77]. Word frequency was not used because of its high correlation with word length (r = -.63; see for more information [78]). We used word length also as a control variable because especially rhyme words are content words and there could be a main effect of word length as it is one of the most important predictor.

### Stimuli

**Figure 1:**
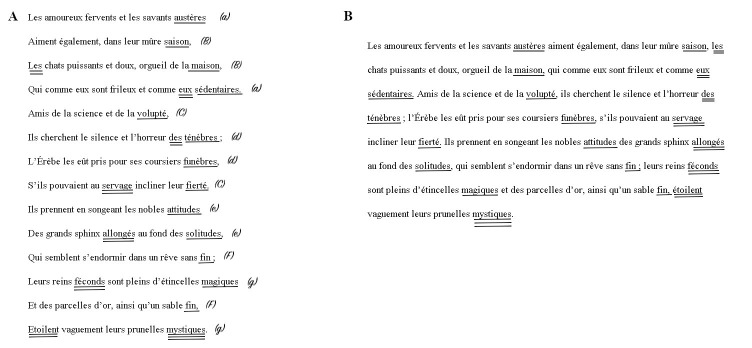
Figure 1. Verse (A) vs. prose presentation (B) of the sonnet *Les Chats*. Note: Final words of a line in the verse presentation, i.e. rhyme words, are underlined once (coded as 1 in the variable Verse-Last Words), final words of a line in the prose presentation are underlined twice (coded as 1 in the variable Prose-Last Words. Letters in parentheses in A indicate the rhyme scheme.

Baudelaire’s poem *Les Chats* is the 56th poem of the collection *Les Fleurs du mal* *(‘The Flowers of Evil’*, [31]). It belongs to the first part of the collection called *Spleen et Idéal* *(‘Spleen and ideal’)* which contains 85 poems including 72 sonnets. This sonnet does not respect a classical French sonnet structure as it presents seven rhyme schemes arranged as: aBBa CddC eeF gFg. It has a French alexandrine verse structure, cut in three sentences and divided into two quatrains and two tercets [1]. For the Visual Presentation of the prose version we first set a maximum page width of 22.62°. Line brakes were chosen following the rule that none of the final words from the verse version should appear in the final word position in the prose version (and vice versa). Both presentation modes are depicted in Figure 1, final words from the verse version are underlined with a single line, final words from the prose version are underlined with a double line.

The general text features are the following: the poem contains 112 words (47 content words, for data analysis we only used 107 regions of interest in cases where two words were not separated with a space as in reflexive pronouns like ‘s’endormir’).

### Procedure

The experiment was conducted in a dimly lit and sound-attenuated room. Data acquisition for each reading (verse or prose) was split in two parts: a first initial reading of the sonnet with eye tracking and a following paper-pencil memory test accompanied by several rating questions and marking tasks. A counterbalanced design was used for the presentation mode to avoid an order effect with half of the subjects first reading the prose version and the other half reading the verse version first.

For the initial reading participants were instructed to read the poem attentively and naturally for their own understanding. Prior to the onset of the poem on a given trial, participants were presented with a black dot fixation marker (0.5°), to the left-side boundary (1.8°) of the first word. The poem was presented to the participants automatically, when they fixated on a fixation marker presented left to the first line. Participants read the poem following their own reading speed: they could go back and forth as often as they wanted without time limit. Texts were presented using a variable-width font (Tahoma), with a letter size of 15-point size (approximately 5 mm, 0.5°). In order to facilitate accurate eye tracking a 1.5-line spacing was used. The prose presentation was as followed: on 8 lines, with left alignment, presented in the middle of the screen and covering 27° width and 15° height. The verse presentation was on 14 lines, with left alignment, presented in the middle of the screen and covering 16° width and 26° height.

After the first reading, participants went to another desk to work on a paper-pencil task adapted from Xue et al. [6]. This first questionnaire had altogether 25 questions concerning memory, topic identification, attention, understanding and emotional reactions. It also included marking tasks where participants had to indicate unknown words, keywords and the most beautiful line of the poem. Most of the questions were open answers. After answering the questionnaire, participants went back to the eye tracker to read the other version of the text. Then, afterwards, a second questionnaire with different open questions was applied concerning possible changes in feelings and text understanding. Because of using different questionnaires, after first and second reading, we renounce of a statistical analysis of the ratings as we could not compare verse to prose reading within participants.

Afterwards, participants should indicate which versions between prose and verse they preferred. Then, they had to fill an empathy scale questionnaire ([79] for French version [80], [81]).

At the beginning and end of the experiment, we used a French translation of the German multidimensional mood questionnaire [MDBF 82] to evaluate the participants’ mood state. This questionnaire assesses three bipolar dimensions of subjective feeling (depressed vs. elevated, calmness vs. restlessness, sleepiness vs. wakefulness) on a 7-point rating scale. The mood ratings at the beginning and the end of the experiment indicated no significant mood changes (all t(17)<1).

Altogether, the experiment took about 40 minutes.

### Data analysis

Eye tracking data was preprocessed using the EyeLink Data Viewer (version 1.11.900, http://www.sr-research.com/data-viewer/). Fixations less than 50 milliseconds were either merged with nearby fixations (distance of less than one degree) or removed for further analysis. All trials for prose/verse reading were checked visually, if necessary, drift corrections on the y-axis were implemented manually. Based on the regions of interest defined by the Data Viewer, data was exported on the level of single words. We chose three duration-based parameters as dependent variables: total reading time, first fixation duration, and gaze duration, for each word, participant, and presentation mode. Whereas first fixation duration and gaze duration are thought to reflect early processes related to visual word recognition and orthographic/lexical access, total reading time is a composite measure that captures both early effects of word recognition and identification, and later processes associated with comprehension and interpretation (e.g., [83] [84]). For all three duration-based variables, skipped words were handled as missing values. To test for rereading reflecting primarily later effects associated with comprehension and possible interpretation difficulties, a categorial variable was created coding whether a reader go back to the respective word after initial reading at least once or not.

Inferential statistics were based on word level LLMs with random intercepts for subjects and words using the lme4 package (version 1.1-15; [85]) in the R environment (version 3.4.3, R Core Team, 2017). Values for the temporal eye-movement measures (i.e., first fixation duration, gaze duration, and total reading time) were logarithmised to handle the slightly right skewed distribution. Before calculating the mixed models, all values with more than three standard deviations above or below the individual mean per participant and word were eliminated as extreme values (1.66-1.71 % of the data). Rereading coded as ‘yes’ or ‘no’ were analysed using a logistic linking function [86]. All models included the same fixed effects: Visual Presentation, Verse-Last Words, Prose-Last Words, Reading Session, and Word Length. The continuous predictor Word Length was centralized. All four categorial predictors were contrasted with a 1/-1 weight. The model also included two random effects which are Subjects and Words of the poem.

For the sake of conciseness, only significant tests associated with fixed effects are reported. Fixed effects were analysed with the Anova function in the car package by using Wald F-tests with a Kenward–Roger approximation of degrees of freedom.

In case of significant interactions additional analyses separately for the verse and prose presentation mode were run following the same principles described above.

**Figure 2:**
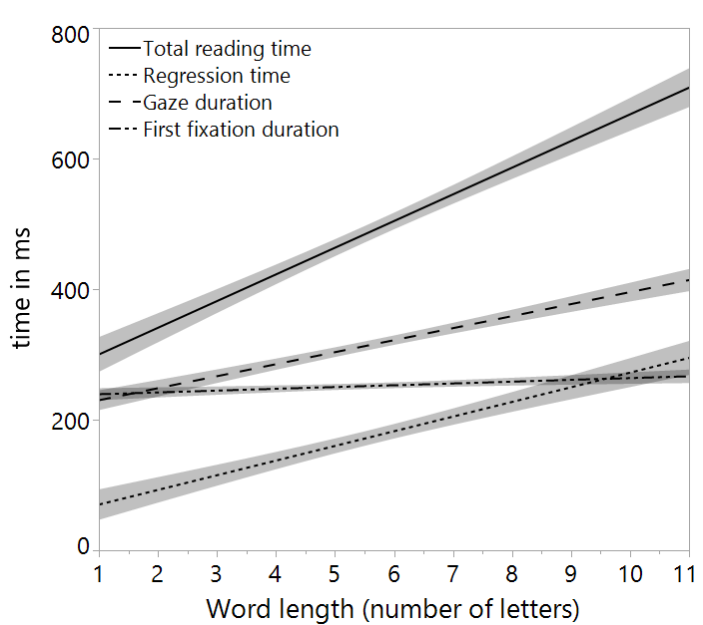
Figure 2. Word Length comparisons (words from 1 to 11 letters) for first fixation duration, gaze duration, total reading time, and rereading time in ms. The shaded area represents 1 SE from the mean. Extreme values were excluded (0.5% of the data).

## Results

### First fixation duration

**Figure 3:**
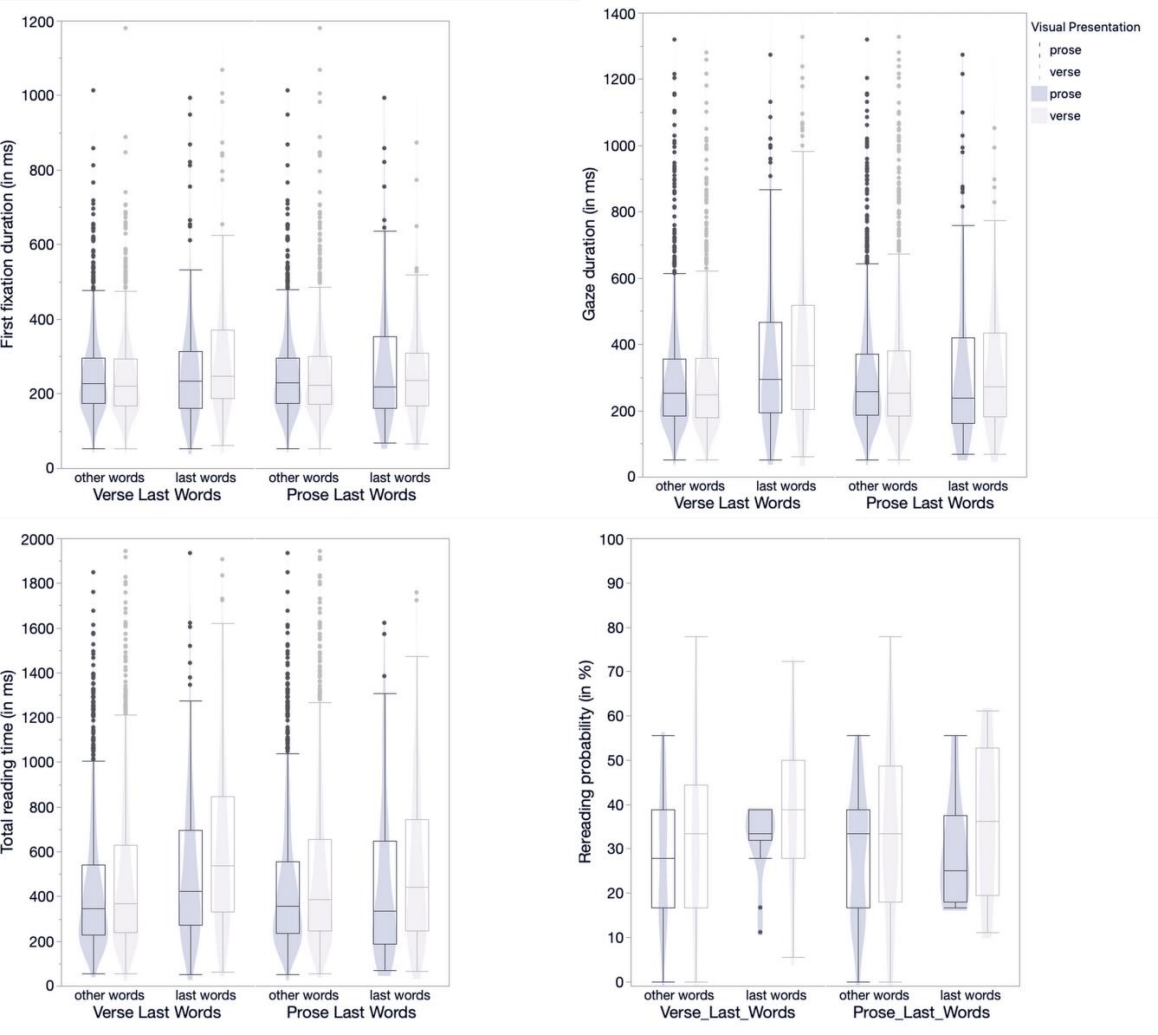
Figure 3. Violin plots and box plots showing the distribution of last words and other words for verse and prose for first fixation duration (top left), gaze duration (top right), total reading time (bottom left), and rereading probability (bottom right). For the three duration-based measures extreme values were excluded (0.5% of the data).

For first fixation duration (cf. Table 1), a significant main effect of Reading Session was obtained (*M _1-reading_* = 257.82; *M _2-reading_* = 245.89): first fixation durations for all words decreased on average for the 2^nd^ session. The missing interaction with the Visual Presentation indicated a general decrease independent of whether participants started with the verse or prose version. Additionally, there was a significant main effect of Verse-Last Word, the variable coding whether a word appeared at the end of a line in the verse version as part of the rhyme scheme of the poem or not. For words pertaining to the rhyme scheme longer first fixation durations were observed compared to the other words (*M_verse last words_* = 285.64, *M_other words_* = 246.33). Moreover, this main effect was qualified by a significant interaction with Visual Presentation. Separate analyses for verse and prose presentation mode (cf. Table 2) indicated that the longer first fixation durations for the Verse-Last Words (i.e., the rhyme words) were observed only in the verse presentation mode (*M _verse last words_* = 305.26, *M_other words_* = 243.95; cf. Figure 3).

Neither the main effects for Prose-Last Words and Visual Presentation nor the interaction between both variables were significant. We also observed no significant effect for Word Length, neither in the overall, nor in the separate analysis (cf. Figure 2).

### Gaze duration

For gaze duration (cf. Table 1), the main effect for Reading Sessions is again a significant with, on average, shorter gaze duration per word for the second compared to the first reading session (*M _1-reading_* = 328.17; *M _2-reading_* = 302.38). Like for first fixation duration, there was no interaction between Reading Sessions and Visual Presentation. Also, the main effect of the Verse-Last Word is significant, indicating longer gaze durations for words pertaining to the rhyme scheme compared to the other words (*M_verse last words_* = 298.67, *M_other words_* = 414.29). As for first fixation duration, a significant interaction between VerseLast word and Visual Presentation could be observed. The separate analysis for verse and prose (cf. Table 3) indicated that the effect for Verse-Last word could be observed only for the verse condition (*M_other words_* = 300.77; *M _verse last words_* = 447.70; cf. Figure 3) but not for the prose condition.

Again, as for first fixation duration neither the main effects for Prose-Last Word and Visual Presentation nor the interaction between both variables were significant. Contrary to the analysis for first fixation duration, we observed a significant main effect for Word Length indicating longer gaze durations for longer words (word length: *M_short_* = 247; *M_medium_* = 313; *M_long_* = 424).

**Table 1:** Mixed model parameters for two duration-based eye tracking measures (first fixation duration and gaze duration) reflecting early processes of visual word recognition and orthographic/lexical access (lexical level).

	First fixation duration			Gaze duration		
	*F*	*ddf*	*p*	*F*	*ddf*	*p*
Visual Presentation	0.05	2904.14	0.82	2.28	2909.40	0.13
Reading Session	9.09	2897.42	<0.01	17.21	2903.50	<.0001
Word Length	<1	100.47	-	45.24	100.32	<.0001
Verse Last Words	8.21	90.98	<0.01	10.32	92.75	<0.001
Prose Last Words	<1	110.78	-	<1	119.86	-
Visual Presentation * Reading Session	<1	16.00	-	<1	16.00	-
Visual Presentation * Verse Last Words	12.45	2891.10	<.0001	5.49	2897.40	<0.05
Visual Presentation * Prose Last Words	<1	2901.54	-	<1	119.86	-

Model: lmer (log(dv) = 1 + Visual Presentation * Verse_Last_Words + Visual Presentation * Prose_Last_Words + Visual Presentation * Reading Session + Word Length + (1|Subjects)+ (1|Words), data_all_, REML=T)

**Table 2:** Mixed model parameters for the separate analysis (verse vs. prose conditions) for first fixation duration reflecting processes of visual word recognition and orthographic/lexical access (lexical level).

	First fixation duration					
	Verse condition			Prose condition		
	*F*	*ddf*	*p*	*F*	*ddf*	*p*
Reading Sessions	1.39	16.00	<.0001	<1	16.00	-
Word Length	<1	99.13	-	<1	100.67	-
Verse - Last Words	16.62	88.94	<.0001	<1	90.15	-
Prose - Last Words	<1	100.90	-	<1	106.55	-

Model: lmer (log(dv) = 1 + Verse_Last_Words + Prose_Last_Words + Reading Session + Word Length + (1|Subjects)+ (1|Words), data, REML=T)

**Table 3:** Table 3. Mixed model parameters for the separate analysis (verse vs. prose conditions) for gaze duration reflecting processes of visual word recognition and orthographic/lexical access (lexical level).

	Gaze duration					
	Verse condition			Prose condition		
	*F*	*ddf*	*p*	*F*	*ddf*	*p*
Reading Sessions	<1	16.00	-	<1	16.00	-
Word Length	20.59	99.39	<.0001	40.30	100.27	<.0001
Verse - Last Words	15.49	91.23	<0.001	1.35	90.77	-
Prose - Last Words	<1	104.38	-	<1	111.20	-

Model: lmer (log(dv) = 1 + Verse_Last_Words + Prose_Last_Words + Reading Session + Word Length + (1|Subjects)+ (1|Words), data, REML=T)

### Total reading time

For total reading time per word (cf. Table 4), the main effect for Visual Presentation was significant. Total reading time per word increased for the verse version compared to the prose one (*M_prose_* = 454.58, *M_verse_* = 525.15). The main effect of Reading Sessions was also significant. (*M_1-reading_* = 543.69; *M_2-reading_* = 434.44). Comparable to first fixation duration and gaze duration, total reading time for each word decreased when reading the sonnet for the second time. Again, the interaction between Visual Presentation and Reading Sessions was not significant, indicating a general facilitation for the second Reading Session irrespective of the order of the Visual Presentation. Also, the main effect for Verse-Last Word was significant indicating longer total reading times for rhyme words compared to all other words (*M_verse last words_* = 616.71, *M_other words_* = 468.90). Again, this main effect was confirmed by a significant interaction between Visual Presentation and Verse-Last Word (cf. Figure 3). The separate analysis for verse and prose condition (see Table 5) showed, that the effect for Verse-Last Word could be observed only in the verse but not in the prose condition. Only when presented in verse form, total reading time for words appearing at the end of a line as part of the rhyme scheme were significantly longer compared to all other words in the sonnet (*M_other words_* = 493.61; *M_verse last words_*= 706.56)

As first fixation duration and gaze duration, neither the main effects for Prose-Last Word nor the interaction between Prose-Last Word and Visual Presentation were significant. According to the analysis for gaze duration, the main effect for Word Length was significant in both the overall models as well as in the two-separate analyses for verse and prose conditions, indicating increased total reading times for longer words (cf. Figure 2).

### Rereading

To analyse possible effects of rereading, i.e. cases when readers return to a word to read it a second or third time, a binary variable was used coding whether rereading was observed or not. By calculating a logistic regression, we observed significant main effects for Visual Presentation and Reading Session. Rereading probability was higher in verse compared to the prose condition (*M_prose_*= 28.29% ; *M_verse_* = 32.71%) as well as in first compared to second reading (*M_1-reading_* = 35.25%, *M_2-reading_* = 25.75%). There was no significant interaction between Visual Presentation and Reading Session. As for total reading time, the main effect for Verse-Last Word was significant. Words appearing at the end of a line in the verse condition received more rereading than all other words (*M_verse last words_* = 34.52%, *M_other words_* = 30.55%; cf. Figure 3). Contrary to all analyses, the interaction between Visual Presentation and Verse-Last Word was not significant which indicates a generally higher rereading probability for rhyme words compared to all other words independent of Visual Presentation mode.

Again, as for all other analyses, neither the main effects for Prose-Last Word nor the interaction between ProseLast Word and Visual Presentation were significant. The main effect for Word Length was significant indicating increasing rereading probability for increasing word length (cf. Figure 2).

**Table 4:** Mixed model parameters for two eye tracking measures reflecting (also) later comprehension processes (lexical level).

	Total reading time			Rereading (yes/no)^1^	
	*F*	*ddf*	*p*	*Chisq*	*p*
Visual Presentation	32.43	2900.38	<.0001	11.48	<.001
Reading Session	86.82	2895.04	<.0001	42.06	<.0001
Word Length	131.41	100.30	<.0001	64.84	<.0001
Verse Last Words	1.73	92.78	0.19	5.46	<.05
Prose Last Words	1.80	121.14	0.18	<1	-
Visual Presentation * Reading Session	<1	16.00	-	<1	-
Visual Presentation *
Verse Last Words	9.83	2888.47	<0.01	<1	-
Reading Session *
Prose Last Words	<1	2899.02	-	<1	-

Notes: ^1^Rereading was coded as a binomial variable and was analysed using a logistic linking function; the random and fixed effects used in both models were equivalent to the model used for first fixation and gaze durations.

**Table 5:** Mixed model parameters for the separate analysis (verse vs. prose conditions) for total reading time (lexical level)

	Total Reading Time					
	Verse condition			Prose condition		
	*F*	*ddf*	*p*	*F*	*ddf*	*p*
Reading Sessions	1.43	16.00	-	1.63	16.00	-
Word Length	100.78	99.06	<.0001	91.95	100.70	<.0001
Verse - Last Words	5.84	90.37	<.01	<1	91.16	-
Prose - Last Words	<1	104.29	-	1.08	109.40	-

Model: lmer (log(dv) = 1 + Verse_Last_Words + Prose_Last_Words + Reading Session + Word Length + (1|Subjects)+ (1|Words), data, REML=T)

## Discussion

We used eye tracking to measure the effect of Visual Presentation mode on reading behavior of a French sonnet. Based on theoretical assumptions of Fabb [7] and empirical work by Hanauer [40], we assumed that presenting a sonnet as a prose text compared to the presentation in its original verse form leads to shorter total reading time per words. Whether this hypothesized effect was due to faster initial processing, reflected in shorter first fixation durations and gaze durations, or due to shallower, more fluent comprehension processes, reflected in fewer rereading, was one of the open questions. Besides this, we wanted to test the interaction between rhyme words and visual presentation mode. As one of the most obvious features of a sonnet, its verse presentation presents a rhyme scheme that coincides with the end of line position. We assumed an interaction between Visual Presentation and final word position in verse form (Verse-Last Words). To control for additional effects, line end position in prose form (ProseLast Words), Word Length and Reading Session were taken into account as control variables.

In line with our first hypothesis, the eye tracking data indicated a significant main effect for Visual Presentation for total reading time and rereading probability. The main effect for total reading time was qualified by a significant interaction between Verse Last Word and Visual Presentation. This interaction was also significant for the two early eye tracking measures, first fixation and gaze duration, but not for rereading. For all duration-based measures, we observed longer reading times concerning rhyme words compared to all other words, but only when presented in the verse condition. As assumed, the effect of Visual Presentation interacts with word position. Only when being a rhyme word and being the last word in a line, we observed longer reading times for these words compared to all other words. In contrast, words presented at the end of a line in the prose condition were read as fast as all other words. No significant main effects or interactions were observed with Prose Last Word. The main effect for Reading Session was always significant indicating faster processing (both early and late) in first compared to second reading, independent of the order of the Visual Presentation mode. A significant effect for Word Length was observed in all measures except for first fixation duration (cf. Figure 2).

The main effect of Visual Presentation was observed only for total reading time. Due to the missing main effect of Visual Presentation for first fixation and gaze duration –the two eye tracking parameters associated with early processes of word identification– we conclude that on average longer total reading times observed in the verse condition are based on rereading differences. The verse Visual Presentation induced more demands for going back in order to link the meaning of specific words or lines, a step necessary to understand rhetorical features divided over several words and/or lines. For example, as Jakobson and Lévi-Strauss [1] pointed out, the first line of the poem presents a double opposition between “lovers” and “scholars”, and “fervent” and “austere”. To process that rhetorical feature, a reader needs to interlink those words. Also, there are specific syntactic patterns that are highlighted by verse Visual Presentation, such as “love too” and “star vaguely”, both verbs and adverbs in French and positioned in the beginning of lines 2 and 14. Our eye tracking data therefore are in line with the assumptions and findings of Fabb [7] and Hanauer [40] that reading the same text in different presentation forms induces different reading patterns. Our results indicated that the different reading patterns are most obvious in the rereading behavior suggesting that not the initial processing, but later, deeper comprehension processes cause these reading patterns. However, whether the longer rereading observed in the verse version is due to a translation process as assumed by Fabb [7] is still an open question. Different reading patterns could also be associated with different reading profiles as described in Kuiken, Campbell and Sopčák [87]. For example, reading the verse version could lead to more meaningful engagement. To understand the reason behind different reading patterns, future studies should focus also on readers’ experience during and after reading.

To link the eye movement patterns with text processing, we assumed that there should be a greater time effect when reading final words in the verse presentation. The significant interaction between Visual Presentation and Verse-Last Words and the results of separate analyses for verse and prose showed that the differences in the processing of rhyme words were only observed in the original verse form. In this verse condition, rhyme words dwelled upon longer, an effect visible in all duration-based eye tracking measures. In the prose condition, rhyme words were presented at all possible line positions (see Figure 1), but never occurred at the final position. Moreover, the words belonging to one rhyme pair did not occur at the same position in a line. Arranged this way, no significant differences in duration-based eye tracking measures were observed. Neither initial processing nor total reading time differed significantly between rhyme words and all other words. We therefore assume that readers identified rhyme pairs basically via regressive eye movements since the main effect for Verse-Last Words was significant also for rereading. This is in touch with findings indicating that readers are sensitive to rhyme schemes [25] [38] [40] [44] [50] [65].

To check other possible interpretations of the observed differences between rhyme words and all other words, we included three additional variables into our models. In general, all rhyme words are content words which are often longer, and more complex than other words used in the poem. We therefore added Word Length as a covariate into all models. As demonstrated by a majority of eye tracking studies, word length is one of the most important predictors for eye tracking behavior (e.g. [76] [88] [89] [90] [91] [92]). We also found a positive relationship between word length and all duration-based eye tracking measures, but no significant effect of word length on first fixation duration. This is also consistent with the literature indicating that word length effects are most prominent in later processing, with increased refixation probability on longer words [93]. The fact that we still observed significant differences between rhyme words and all other words with Word Length as a covariate supports our rhyme scheme interpretation.

To rule out the alternative explanation that longer reading times for final rhyme words are due to so called wrapup processes and/or preparation of the line sweep, we checked whether words presented at the end of a line in the prose condition also differed from all other words. However, we observed no significant effects for Prose-Last word, neither in the full models, nor in the separate analyses conducted for the prose condition. The results are also in line with studies by Slattery and colleagues [94] reporting line swap effects only for the first word of the next line. In total, our results our rhyme scheme interpretation.

Besides the main effect of Visual Presentation, we found significant main effects for Reading Sessions for all four measures. Reading times were always shorter for second compared to first reading. This effect is in line with the well described facilitation effect of rereading often observed in studies using expository texts (see [95] for a review). Recent work by Xue et al. [6] also observed this general facilitation for Shakespeare sonnets. In our study we observed faster initial processing in second compared to first reading visible in shorter first fixation durations and gaze durations. Also, later processing seems to be less demanding during second reading as we observed shorter total reading times and lower rereading probability. As assumed for expository texts, the general facilitation effect relies, at least partially, on the higher familiarity with the text during second reading. Interestingly, the general facilitation for second reading does not interact with the order of presentation. Independently of whether the participants started with the verse or the prose form, second reading was always faster.

Several studies have aimed at understanding how readers adapt to literary text type such as verse or prose [40] [41] [42] [51] [52]. Some evidence points to the fact that readers do a categorization at an initial stage leading to speculations about the type of information used for that initial decision [40] [41] [42] [51] [52]. According to Hanauer, it is formal textual features, i.e. both visual presentation and rhyme scheme. Our findings show that especially the processing of rhyme words differed from that of all other words when the sonnet is presented in its original verse from. These differences in processing occurred right from the beginning, i.e. also in first fixation durations. Therefore, it can be assumed that the initial categorization and decision for a reading style is built on information about the overall visual text form, with the recognition of rhyme being used as a confirmation for that initial decision. Nevertheless, readers also seem able to adapt the initially chosen reading style when detecting some ‘inconsistencies’. Presenting a sonnet as a prose text does not turn it into a prototypical prose text, since poetic features like rhyme or the higher number of rhetorical figures are still kept intact. Thus, our readers still detected rhyme words in the prose presentation, but processing differences were visible only in rereading probability. This suggests that readers in the prose condition adopt the initially chosen reading style when recognizing poetic text features. In terms of the NCPM [2] [12] this could mean that they (temporarily) switch from the upper ‘prose reading’ to the lower ‘poetry reading’ route.

Our effects are well in line with the assumption of space theory [55] [58] which encompasses all space features of a text, between words and margins. In poetry, space management is a central feature deliberately shaped by the poet. As highlighted by Derrida [96], “spacing” is an active, productive characteristic of space which could become a medium of communication. While West-Pavlov, the main advocate of space theory, did not consider the reader in its theory, other scholars, such as La Charité [56] considered both readers and eye movements. He proposed that space management could guide gaze path to build sentence semantics. Indeed, the prose visual presentation does not take into account space managing. In general, for reading prose, space is not helpful for meaning construction as words are more or less randomly presented on a page [57] [96]. The fact that the initial processing of rhyme words differed from other words only in verse but not in prose thus may be a result of spacing. Our results are also in line with the idea that spacing can be interpreted as a kind of punctuation [53], creating wrap- up-like effects and processing pauses as seen in punctuation studies ([97], see also [94] [98]).

The eye movement patterns observed in the present study are also in line with the assumed role of top-down expectation effects [40] [52]. According to these authors, expectations as a consequence of the initial decision about a reading strategy influence the allocation of attention to different elements of the text. Moreover, depending on the visual presentation, features of the text are more or less salient and alter the available cues [40] [99]. Especially in verse, the presentation of rhyme words at the end of a line attracts attention to these words visible in longer processing times. This effect could also be interpreted as a reading strategy [40]. The source of different reading strategies associated with expectation differences is still unknown. Hanauer [40] speculated that cultural education may be responsible for these behavioral changes while Miall and Kuiken [99] asked what is part of the education and what would be a part of an easier and more natural way to understand poetry. To disentangle education effects from other possible sources, one would need more participants with a wider range of cultural-educational differences.

With regard to a key assumption of the NCPM [8] [9] [12] namely that longer processing times are linked to a higher proportion of FG, our results show that processing time and reading style not only depend on text features like the FG/BG quotient. At the text level, the amount of FG and BG features is the same for both presentation conditions, but we observe significant differences in reading behavior. Thus, reading behavior is the result of the interaction between reader, text, and context (here the way the text is presented). This interaction is present in the model but deserves more elaboration in a future version. Of course, replication studies using other poems confirming our findings are needed to motivate such model revisions. Furthermore, future studies could try to quantify the FG/BG quotient of poems to allow more detailed predictions e.g. on eye movement behavior. One track could be the use of qualitative-quantitative narrative analysis (Q2NA) tools [4] like the Abstractness Scale [2] or the Foregrounding Assessment Matrix [100].

**Ethics and Conflict of interest **

We declare that the contents of the article are in agreement with the ethics described in http://biblio.unibe.ch/portale/elibrary/BOP/jemr/ethics.html and that there is no conflict of interest regarding the publication of this paper.

**Acknowledgements**

This research was supported by CIERA, Erasmus+ program, and IFRA/SHS.

We wish to thank specifically Teresa Sylvester for her help and remarks.
